# Predictors of endobronchial forceps utilization for inferior vena cava filter retrieval: when snare retrieval fails

**DOI:** 10.1186/s42155-023-00392-9

**Published:** 2023-11-11

**Authors:** Richard D. Kang, Philip Schuchardt, Jonathan Charles, Premsai Kumar, Elena Drews, Stephanie Kazi, Andres DePalma, Adam Fang, Aislynn Raymond, Cliff Davis, Kamal Massis, Glenn Hoots, Rahul Mhaskar, Nariman Nezami, Jamil Shaikh

**Affiliations:** 1 University of South Florida, Morsani College of Medicine, Tampa, FL USA; 2 Department of Radiology, University of South Florida Health, Tampa General Hospital, Tampa, FL USA; 3 Department of Radiology and Image Guided Medicine, School of Medicine, Emory University, Atlanta, GA USA; 4 Division of Vascular and Interventional Radiology, Department of Diagnostic Radiology and Nuclear Medicine, University of Maryland School of Medicine, Baltimore, MD USA; 5 Radiology Associates of Florida, Tampa, FL USA; 6 Experimental Therapeutics Program, University of Maryland Marlene and Stewart Greenebaum Comprehensive Cancer Center, Baltimore, MD USA

**Keywords:** Inferior vena cava filter retrieval, Endobronchial forceps, Snare failure, Hook embedment, Strut penetration

## Abstract

**Background:**

Endobronchial forceps are commonly used for complex IVC filter removal and after initial attempts at IVC filter retrieval with a snare have failed. Currently, there are no clear guidelines to help distinguish cases where primary removal should be attempted with standard snare technique or whether attempts at removal should directly be started with forceps. This study is aimed to identify clinical and imaging predictors of snare failure which necessitate conversion to endobronchial forceps.

**Methods:**

Retrospective analysis of 543 patients who underwent IVC filter retrievals were performed at three large quaternary care centers from Jan 2015 to Jan 2022. Patient demographics and IVC filter characteristics on cross-sectional images (degree of tilt, hook embedment, and strut penetration, etc.) were reviewed. Binary multivariate logistic regression was used to identify predictors of IVC filter retrieval where snare retrieval would fail.

**Results:**

Thirty seven percent of the patients (*n* = 203) necessitated utilization of endobronchial forceps. IVC filter hook embedment (OR:4.55; 95%CI: 1.74–11.87; *p* = 0.002) and strut penetration (OR: 56.46; 95% CI 20.2–157.7; *p* = 0.001) were predictors of snare failure. In contrast, total dwell time, BMI, and degree of filter tilt were not associated with snare failure. Intraprocedural conversion from snare to endobronchial forceps was significantly associated with increased contrast volume, radiation dose, and total procedure times (*p* < 0.05).

**Conclusion:**

IVC filter hook embedment and strut penetration were predictors of snare retrieval failure. Intraprocedural conversion from snare to endobronchial forceps increased contrast volume, radiation dose, and total procedure time. When either hook embedment or strut penetration is present on pre-procedural cross-sectional images, IVC filter retrieval should be initiated using endobronchial forceps.

**Level of evidence:**

Level 3, large multicenter retrospective cohort.

## Background

Inferior vena cava (IVC) filter placement is indicated for pulmonary embolism (PE) prophylaxis in patients with deep venous thrombosis (DVT) and contraindications to standard anticoagulation therapy (AC) [1]. However, recent U.S. Food and Drug Administration (FDA) guidelines recommend prompt retrieval of IVC filters once patients are no longer at risk of thrombus formation or embolization [2–4]. Despite prior research demonstrating a positive correlation between IVC filter dwell time and risk of adverse events (i.e., IVC filter fracture, perforation, and migration), national retrieval rates have historically remained low [5–10]. From 1999 to 2008, the reported number of IVC filters placements had approximately doubled, but only an estimated 1.2 to 5.1% of IVC filters were retrieved in 2008 [10]. In a more recent report, the overall IVC filter retrieval rate was estimated at approximately 6.6% during the ten-year period from 2004–2014 [6]. These low rates of IVC filter retrieval have been attributed to several factors including lack of proper follow-up or dedicated programs to ensure timely IVC filter retrieval [11–17]. In addition to higher risk of IVC filter malfunction or complications, prolonged dwell time has also been associated with increased risk of snare retrieval failure [2, 5]. A recent systematic review estimated snare retrieval failure rates as high as 60% [18]. In the recent decade, more advanced IVC filter retrieval techniques, such as endobronchial forceps mediated retrieval have become a mainstay for complex removals [19]. While other advanced retrieval techniques such as balloon displacement, sling, or dissection, have been developed to address deficiencies with snare retrieval, utilization of endobronchial forceps has been gaining popularity for its efficacy and safety [19–26]. For example, our large retrospective study showed endobronchial forceps had an overall success rate of 98.8% [17]. In our current review of the literature, clinical guidelines for direct utilization of endobronchial forceps seems to be lacking. While several factors such as degree of tilt, dwell time, and hook embedment have been reported to increase IVC filter retrieval difficulty, a trial-and-error approach is often employed for conversion of snare to more advanced IVC filter retrieval techniques, such as use of endobronchial forceps [27–32]. While escalation to endobronchial forceps is typically considered only after snare failure, we surmised that this clinical approach could potentially increase procedural times, radiation exposure, and place the patient at higher risk for complications. The aim of this study was to identify the clinical and imaging indications for direct utilization of endobronchial forceps, bypassing any attempts with a snare.

## Methods

### BStudy design and population

This was a retrospective study of patients with existing IVC filter referred to our institutions for IVC filter retrieval from January 2015 to January 2022. The IRB protocol was reviewed and approved by University of South Florida IRB committee. All subjects provided written informed consent. Eligibility for retrieval was periodically evaluated with chart review and clinical assessment of the primary physician’s consultation. When retrieval was indicated, patients were seen in clinic to discuss the benefits and risks associated with retrieval prior to scheduling the procedure. Additional referrals for filter retrievals were placed by outside institutions or inpatient consultations for complex retrievals. Patient who had concurrent ileocaval thrombosis, complications intraprocedurally, who required piece-meal filter retrieval, or those with permanent filters were not included in this study. Additionally, procedures with complications were not used in this study as the purpose was to evaluate only predictors of successful removal. Furthermore, retrievals which required use of adjunctive techniques (i.e., loop snare/hangman technique) or other advanced retrieval techniques (i.e., laser sheath, balloon disruption/displacement) were removed from the study. All filters were removed via neck access in the right internal jugular vein, right external jugular vein or right subclavian vein. Variables Data on patient characteristics including age/sex, BMI, filter tilt, concurrent anticoagulation use, filter dwell time, success with either snare or endobronchial forceps were collected from a retrospective database. Additionally, filter characteristics including filter type, migration, fracture, strut penetration, filter hook encapsulation/embedment and pericaval organ involvement were recorded.

### IVC filter retrieval procedures

All retrieval procedures were performed by board-certified interventional radiologists with similar experience. Filter retrieval was attempted when mechanical caval prophylaxis from lower extremity deep venous thrombosis was no longer indicated. All cases were technically successful; which the authors defined as en bloc retrieval of the IVC filter. Snare retrieval techniques were initially attempted by utilizing either Argon Medical (Argon Medical, Athens, TX) or the GuntherTulip (Cook Medical, Bloomington, IN) IVC filter retrieval kits. Alternatively, snare retrieval was also attempted with use of coaxial inner and outer vascular sheaths (Flexor; Cook Medical, Bloomington, IN) with a gooseneck endovascular snare device (12- to 20-mm EnSnare; Merit Medical Systems, Inc, South Jordan, UT). The retrieval kit or device was left to operator preference and ultimately with their comfort of use. When snare retrieval failed, advanced techniques were employed via a 16-20F vascular sheath and use of rigid endobronchial forceps (Lymol Medical, Woburn, MA).

### Statistical analysis

Data was analyzed by statistical software (IBM SPSS statistics ver. 27, Chicago, IL, USA). Frequencies are reported for categorical data and median/range for continuous data. Categorical data was analyzed via the Pearson Chi-square test and Fischer's exact test. Binary multivariate logistic regression was used to investigate the association of clinical data with successful retrieval via either standard or advanced techniques. The Hosmer and Lemeshow (HL) Test was used to test the model goodness of fit. Data were considered significant for *p* < 0.05.

## Results

A total of 543 successful IVC filter retrievals from three separate institutions were reviewed. Approximately 48% of the total patient population was female, average age was 55 years (range 18–94 years), and 31% of patients had BMI greater than 30. The three most common indications for IVC filter placement among this cohort were DVT with contraindications to AC (297/543; 54.7%) with PE with contraindication to AC (161/543; 29.7%) and pre surgical prophylaxis (56/503; 10.3%). Standard retrieval was successful in 340 cases (62.6%). Chi-square analysis demonstrated that neither age, sex, filter indication, nor BMI were significantly associated with snare retrieval failure.

The average filter dwell time was noted to be 983 days and 23.4% of patients had filters with dwell times greater than four years. We noted significant association between dwell time greater than four years and snare retrieval failure (*p* < 0.001).

IVC filter tilt was present in 31% of all cases. Upon further subgroup analysis, only 27% of filters retrieved by snare were tilted compared to 72.9% of filters requiring endobronchial forceps (*p* = 0.023). Additionally, filters with greater degree of tilt were associated with failure of snare retrieval (*p* = 0.37). Filters demonstrating either proximal hook embedment into the wall of the IVC (*p* = 0.002) or strut penetration outside the IVC (*p* < 0.001) were associated with snare retrieval failure. The data for tilt, proximal hook embedment, and strut penetration are outlined in Table 1. Cases which required conversion to endobronchial forceps were associated with significantly higher total procedural times (*p* = 0.019), contrast use (0 < 0.001), and total absorbed radiation (*p* < 0.001) see Table 2

**Table 1:** Frequency of filter tilt, hook embedment, and strut penetration

Filter Name	Frequency of Filter Tilt (%)	Frequency of Proximal Hook Embedment (%)	Frequency of Strut Penetrating IVC Wall (%)
Celect	14.1	12.6	16.7
Denali	25.3	25.9	18.3
Eclipse	0.0	0.0	0.0
G2	0.0	0.7	0.0
Gunther	34.1	33.6	39.2
Meridian	0.0	0.0	0.0
Option	21.8	22.4	24.2
Recover	1.2	1.4	0.0

**Table 2:** Intraprocedural variables

Intraprocedural Variables	Snare Retrieval	Forceps Retrieval
Average Fluoroscopy Time (sec)	422	685
Average Radiation Exposure (mGy)	103	1678
Average Contrast Use (mL)	32	65

Binary logistical regression was performed to develop a predictive model in which a clinical scenario would outline when snare retrieval would likely fail (Table 3). When adjusted for age and gender, the presence of the proximal hook embedment within the IVC wall (OR: 4.55; 95% CI 1.74–11.88; *p* = 0.002) and struts penetrating outside the wall (OR: 56.46; 95% CI: 20.21–157.71; *p* = 0.001) were independent factors that predicted snare retrieval failure. In total, 49 filters demonstrated filter strut penetration into surrounding visceral organs or vascular structures (Table 4). These cases were excluded from the predictive model because successful retrieval was seen only with endobronchial forceps. The presence of tilt (OR 2.00; (95% CI: 0.44–9.13; *p* = 0.370), angle of the tilt (OR: 1.06; (95% CI: 0.88–1.27; *p* = 0.570), and dwell time (OR: 0.46; 95% CI: 0.09- 2.34; *p* = 0.719) were not statistically significant predictors of snare retrieval failure within this model.

**Table 3:** Predictive factors of snare retrieval failure

Characteristics	Multivariate analysis Odds Ratio (95% confidence intervals)	*P*-value
BMI (> 30)	1.05(0.46–2.38)	0.901
Filter Dwell Time > 4 years	0.46(0.09–2.34)	0.352
Filter Tilt	2.00(0.44–9.13)	0.370
Angle of Filter Tilt	1.06(0.88–1.27)	0.570
Proximal Hook Embedded in Wall	4.55(1.74–11.88)	**0.002**
Strut Penetration Outside Wall	56.46(20.21–157.71)	** < 0.001**

**Table 4:** Complex filter lie and penetration

Penetration into Organs around IVC	Number of Cases (% of Total)
Struts in Small Bowel	20 (3.6%)
Struts in Aorta	6 (1.1%)
Struts in Renal Vein	5 (0.9%)
Struts in Renal Artery	2 (0.3%)
Struts in Vertebral Body	16 (2.9%)

The absolute numbers and frequency of filters penetrating adjacent vessels or organs is included in Table 4. All filters exhibiting these characteristics required utilization of endobronchial forceps.

## Discussion

Over the past several decades, IVC filter placement has increased at a rapid pace [9, 33]. A retrospective cross-sectional study noted that filter placements more than doubled between the years 2000 and 2009 [34]. While one retrospective study examining Medicare data from 2012 to 2015 reported an increasing trend for filter retrieval, the overall IVC filter retrieval rates remain historically low [6, 7, 35]. Despite appropriate clinical indications for filter retrieval, many patients are lost to follow up [11–17]. Several studies have reported improvements in retrieval rates following implementation of IVC filter clinics, filter tracking, and scheduling programs. However, estimated retrieval rates remain suboptimal at approximately 30% [36, 37]. Unfortunately, these filters with prolonged dwell times have been associated with increased risk of complications and necessity of advanced retrieval techniques (not limited to use of endobronchial forceps) [2, 5, 37]. Visceral organ strut penetration is a known complication of IVC filters, and at our institution we take necessary steps to adequately manage these patients. Those with gastrointestinal organ (stomach, small bowel) strut penetration receive a full bowel preparation so to minimize the risk of the patient developing bacteremia, as well as placement on prophylactic antibiotics. In those cases with renal artery or vein puncture as demonstrated on pre-removal imaging, we perform a pre-removal and post-removal arteriogram/venogram to demonstrate the level of vessel injury. This management holds for any vessel puncture. Past research has shown advanced techniques (i.e., endobronchial forceps, wire loop, and laser sheath) to be quite effective, with retrieval rates ranging from 90 to 100% [26, 38–44]. However, several single-center studies have reported an association between advanced techniques and increased risk of complications [17, 43, 45]. In a retrospective study at our institution, we observed a significant increase in complication rates for endobronchial forceps compared to snare retrieval (14.5% vs 1.7%, respectively) [17]. These confounding factors likely existed as these patients had significantly increased dwell times, fractured struts and typically presented with ileocaval thrombosis. Furthermore, for this work, stratification of the variables based on commercial filter type can be found here [reference: Shaikh, J., et al. *Predictors For Use of Forceps Directed Inferior Vena Cava Filter Retrieval: When Standard Techniques Fail*. CIRSE 2022) This stratification analysis should require its own full manuscript and future work is underway for this. In contrast, a meta-analysis by Merritt et al. revealed no significant difference between snare retrieval and advanced retrieval techniques in terms of safety and efficacy [41]. Currently, the clinical indications for direct utilization of advanced techniques remains unclear. In 2021, Giurazza et al. proposed a two-score system to gauge the necessity of advanced techniques using a “complexity score” and an “outcome score” with positive predictive values of 64.7% and 50%, respectively [28]. Other research has focused on specific parameters such as angle of tilt, but results have been inconsistent. For example, a single-center retrospective study of filter retrievals from 2015 to 2017 noted that lateral tilt was not predictive of the need for advanced techniques. In contrast, two other single-center retrospective studies found a significant positive association between tilt and snare retrieval failure [27, 30, 39]. The purpose of our study was to shed further light on these issues and identify clinical factors which are strongly predictive of snare retrieval failure. We considered variables such as dwell time, and filter tilt/angle, but found that only proximal hook embedment (*p* = 0.002) and strut penetration (*p* = 0.001) were significant predictors for snare retrieval failure. When either of these factors are noted on pre-procedural imaging, it is recommended to defer snare retrieval and directly utilize endobronchial forceps. One tangible benefit of our predictive model may be a reduction in intraprocedural times and radiation exposure. We found significant increases in total procedural times, contrast use, and total absorbed radiation for cases requiring utilization of endobronchial forceps. We noted that none of these cases required venous stenting or demonstrated concurrent ileocaval occlusion, which could act as confounders. Although further studies are warranted, we would expect a decrease in intraprocedural time, anesthesia use, contrast use and radiation exposure had initial snare retrieval been deferred. Furthermore, utilization of our predictive model may decrease complication rates due to possibility of filter migration or strut fracture during snare retrieval failure. A single-center retrospective study in 2019 compared 378 standard and 89 advanced retrievals and noted a significant decrease in fluoroscopy time and radiation exposure but increased incidence of complications and use of general anesthesia [42]. However, this study did not differentiate between the various advanced techniques (i.e., endobronchial forceps, balloon displacement, laser sheath, etc.) which may be a direction of future research. Our predictive model may also be helpful to interventionists who are not comfortable with utilization of endobronchial forceps or who work in settings lacking the necessary resources required for utilization of endobronchial forceps. If either hook embedment or strut penetration is observed, snare retrieval will likely fail, and interventionists can refer these patients to centers of excellence. Our model can be used to guide treatment algorithms with the goal of improving patient care, decreasing costs and minimizing complications. There are several limitations to this study. Utilization of endobronchial forceps was our institution’s preferred method of advanced filter retrieval. The use of this specific advanced technique was based on operator and institutional preference. We did not investigate use of adjunctive techniques (i.e., loop snare/hangman technique) or other advanced retrieval techniques (i.e., laser sheath, balloon disruption/displacement). The study was carried out at large quaternary care academic centers on the east coast and did not include interventionalists who practice in smaller settings thereby limiting the broader clinical implications of our findings. Advanced IVC filter retrieval often requires complex techniques which should optimally be performed in centers with high level of expertise in these procedures. Finally, multiple types of IVC filters were encountered during the study period. Technical challenges encountered during filter retrieval may vary by device. A subgroup analysis of our cohort demonstrated associations between filter type and tilt, penetration, etc. However, we felt inclusion of this data into the predictive model would lead to inaccuracies as the filters were not tested under equal conditions and sample size for each type of filter varied significantly. Future studies will be necessary to identify clinical predictors for snare retrieval failure based on filter type. Additionally, future work will work to measure to rates of filter complication based on filter type, including vessel and visceral organ penetration. Furthermore, the impact of bypassing snare retrieval on intraprocedural variables (i.e., time, complications, and radiation exposure), healthcare costs, and complication rates will also need to be explored in future studies.

## Conclusions

Based on our prediction model, hook embedment within the IVC wall and strut penetration outside the vessel wall were strongly predictive of snare retrieval failure. Determining these clinical variables on pre-procedural imaging may guide referral of patients to centers with expertise in use of endobronchial forceps where IVC filters with complex lie can be safely removed. Incorporation of our study findings may potentially lead to a reduction in procedural times, incidence of complications and overall healthcare costs.
